# History of the Canadian Medical Education Journal

**DOI:** 10.36834/cmej.69944

**Published:** 2020-03-16

**Authors:** Claudio Violato, Marcel F. D’Eon

**Affiliations:** 1 Universtiy of Minnesota, Minnesota, USA; 2 University of Saskatchewan, Saskatchewan, Canada

## Part 1: pre-2010-2013 (Claudio Violato, founding editor)

It is a pleasure to write on the “early history” of the *Canadian Medical Education Journal* on its tenth anniversary. In the editorial for the inaugural issue (March 2010), we wrote that we embarked on this adventure with some trepidation because of the many challenges of starting a new journal.^1^These included establishing an editorial board, needing high quality submissions, seeking help from expert peer reviewers, and working hard for manuscript selection, preparation and distribution of our issues. At that time, while there was some interest and support for such an ambitious undertaking, it was hard to mobilize.

By the *fin de siècle* (20^th^ century), we believed that Canadians were among the leaders in medical education and contributed substantially to the scholarly and research enterprise. Subsequent research has confirmed this: Canadians had and may still have the highest proportional publication productivity in medical education in the world.^2^It seemed peculiar, therefore, that there was not a uniquely Canadian forum (a journal) for this scholarly activity.

Medical education research expanded rapidly in Canada in the past several decades. For some time, there have been centres at McGill, Toronto, University of Ottawa, Queens, and McMaster, while more recent centres have been established at the Universities of British Columbia, Western Ontario, Alberta and Calgary. These centres undertake foundational research and engage in all forms of scholarship in medical education to produce high quality publications. Many of them train graduate students at the Masters and PhD levels.^2^^-^^10^

By 2007 my colleague, Dr. Tyrone Donnon, and I decided that we would lead the way to a new Canadian journal in medical education. We approached many of the major medical education organizations in Canada (the *Association of Faculties of Medicine of Canada*, the *Canadian Association for Medical Education*, the *College of Family Physicians Canada*, and the *Royal College of Physicians and Surgeons of Canada*, the *Medical Council of Canada*) and many of our colleagues at the major university centers. After about two years of meetings, discussions, business plans, vision and mission statements there were still some unresolved questions and we did not seem to be much closer to our goal. We took another approach: “If you build it, they will come.”^11^

We found some friendly, competent librarians at the University of Calgary who helped us to set up the platform for online publishing, guided us through the process of registering a URL, and provided some initial funding through the Library’s support for publications. With Dr. Donnon as the Associate Editor, me as the Chief Editor, and one of our graduate assistants as a Managing Editor, we launched the CMEJ. We announced the new journal, put out a call for papers, received manuscripts and sent them for review, made editorial decisions, and published the first edition. Getting the next few issues out was very demanding as the three of us managed the whole process. Notwithstanding the formidable challenges of starting a new journal, we built it, recruited more valuable help, persisted, and made it a success.

## Part 2:2014-2019 (Marcel D’Eon, Chief Editor)

I remember the long phone conversation I had with Dr. Jocelyn Lockyer in February 2014. The founding editor, Dr. Violato, had just departed the University of Calgary to work in the US. The CMEJ needed an editor and there did not seem to be anyone readily available to pick it up. I had been an associate editor with the CMEJ for about a year and with the Canadian Journal for the Scholarship of Teaching and Learning for maybe two. I had no idea how to run a journal (does anyone before they start?), but, as we talked, we realized that it would be easier to keep the CMEJ alive (even faintly) than to try to revive it once dormant (or dead). I volunteered. It was a brave but naïve move. With lots of help from Tina Voth, the manager, Dr. Lockyer, Senior Associate Dean at the Cumming School of Medicine, University of Calgary, and with most of the associate editors that had been previously recruited, we continued the CMEJ adventure.

In those first few months, the associate editors embraced the challenge and I recruited others to help with our work. In December 2014, at the last possible opportunity, we managed to publish one issue for 2014, (having published two each year since 2010). We then published two issues in 2015, three in 2016 and then four in each of 2017, 2018, and 2019. We went from about 90 pages per year to now over 400 pages per year. In the spring of 2017, we began to publish images with each issue, a beautiful addition to adorn our web pages. Most of these images arefrom Canadian artists having been displayed at WhiteCoat, Warm (He)Art at CCME.

Tina Voth retired, so we hired our own local talent. We were very fortunate that Dr. Jennifer O’Brien was interested and available to do some part time work for us. She became our Managing Editor in 2014 and has been a force on the CMEJ ever since. We hired a copyeditor and then a support person to help us keep track of the flow of submissions and peer reviews. We improved our processing speed from an average of over 200 days to the first decision following peer review to well under 100 days. We cut in half the average number of days to publication from just under 300 to about 150. We accomplished this even as we grew from an average of 25 submissions per year to over 150—a six-fold increase.

The CMEJ is indebted to our funders. Without their material support we would not have been able to accomplish what we did and may have had to shut down our presses. The University of Calgary helped us out in 2014 with about $10,000 as we transitioned our operation to the University of Saskatchewan which then awarded us a total of $14,000 in publication grants over the next four years. The CAME Foundation provided the CMEJ with over $12,000 in the span of two years. AFMC also generously provided $5000 to top up our operating funds. Finally, in March 2018, the Social Sciences and Humanities Research Council awarded the CMEJ about $87,000 over three years for operational expenses and special projects (translating abstracts and titles and celebrating our 10^th^ anniversary at CCME 2020). An additional “shout out” goes to the University of Saskatchewan that provided salary support for me in my role as Chief Editor that allowed me to edit the CMEJ “at work.” We are so very grateful to the people and organizations who had faith in the CMEJ and continue to invest time and money in our future.

We re-organized our editorial board and recruited many new associate editors from across the country. They represent 12 Canadian medical schools at 13 universities. The associate editors are the people that help screen submissions, assign reviewers, recommend decisions to the Editor, and communicate with the authors. They are the engine that drives the CMEJ. We decided that they should have a role in shaping editorial policy. We therefore made them all members of the editorial advisory board and the editorial advisory board consists mostly of associate editors.

At first, the editorial board met a few times each year over the phone. These were important and helpful meetings. Our first face-to-face meeting took place over lunch in Winnipeg on Tuesday, the last day of the CCME 2016. Strictly BYOCC (Bring Your Own Credit Card), we have met at CCME each year since and will continue to do so. The face-to-face meetings are excellent for team cohesion and dealing with significant editorial issues.

Behind the obvious academic activity of peer reviewing and publishing articles (and some declining – sorry), a national consortium of medical education organizations was forming into what has become the CMEJ Management Board. Meetings followed by more meetings led to consultations and more consultations. Eventually a consensus emerged and a five-partner team with advisors and support from across the country came together to take a leadership role overseeing the business and financial aspects of the CMEJ. There are representatives from the *Association of Faculties of Medicine Canada*, the *Canadian Association for Medical Education*, the *College of Family Physicians Canada*, the *Medical Council of Canada*, and the *Royal College of Physicians and Surgeons Canada*. The co-chairs are affiliated with AFMC and CAME. We are excited that the five national partners that support and run CCME each year are also deeply involved with and supportive of the CMEJ.

## Conclusion (Drs. Violato and D’Eon)

We saw the need for a new forum for the publication and distribution of work from researchers and scholars in Canada and internationally. We built it and as we had hoped, “they” did come. The CMEJ has grown a great deal since those early days. It has taken many people working hard, all of them integral to the journey, to bring us to this place over 10 years later. It will now take many more hands to propel us forward as we become a vital partner in medical education in Canada and then, naturally, across the globe.

## Partie 1 Avant les années 2010-2013 (Claudio Violato, rédacteur fondateur)

J'ai le plaisir d’écrire quelques lignes sur les débuts du *Revue canadienne de l’éducation médicale* continue à l’occasion du 10^e^ anniversaire de sa création. Dans l’éditorial du premier numéro (mars 2010), on disait qu’on commençait notre aventure avec une certaine appréhension parce que la publication d’une nouvelle revue représente de nombreux défis à relever^1^. Il fallait entre autres créer un comité de rédaction, recevoir des articles de grande qualité, solliciter l’aide de pairs évaluateurs, prévoir du temps pour la sélection des manuscrits, la préparation et la diffusion des numéros de la revue. Une entreprise aussi audacieuse suscitait un certain intérêt, mais la mobilisation restait difficile.

Au tournant du 20^e^ siècle, on disait que les Canadiens comptaient parmi les chefs de file du domaine de la formation médicale et qu’ils contribuaient largement aux activités de recherche. Des recherches ultérieures ont permis de confirmer ceci : toutes proportions gardées, les Canadiens arrivaient et continuent d’arriver en tête pour ce qui est de la production de publications dans le domaine de l’éducation médicale dans le monde.^2^ Mais étrangement, il n’existait aucune tribune canadienne (revue) consacrée à cette activité universitaire.

La recherche en éducationmédicale s’est beaucoup développée au Canada au cours des dernières décennies. Depuisquelques années, des centres ont vu le jour à l’Université McGill, à l’Université de Toronto, à l’Université d’Ottawa, à l’Université Queens et à l’Université McMaster. Tout récemment, des centres ont été inaugurés à l’Université de la Colombie-Britannique, à l’Université Western Ontario, à l’Université de l’Alberta et à l’Université de Calgary. Ces centres font de la recherche fondamentale et offrent toutes sortes de bourses d’études en éducationmédicale pour permettre la production de publications de grande qualité. Bon nombre d’entre eux forment des étudiants à la maîtrise et au doctorat.^2^^-^^10^

En 2007, mon collègue, le D^r^ Tyrone Donnon, et moi-même avons décidé de tracer la voie pour la production d’une nouvelle revue canadienne consacrée à l’éducation médicale. On a sollicité l’aide de nombreux organismes canadiens de formation médicale (*Association des facultés de médecine du Canada*, *Association canadienne pour l’éducation médicale,Collège des médecins de famille du Canada,Collège royal des médecins et des chirurgiens du Canada* et *Conseil médical du Canada*) et demandé l’assistance de nombreux de nos collègues travaillant dans de grands centres universitaires. Après environ deux ans de rencontres, de discussions, de plans d’affaires, d’énoncés de vision et de mission, il restait des questions non résolues et on ne semblait pas s’être rapprochés de notre objectif. On a donc choisi une autre formule : « Construisez, et « ils » viendront. »^11^

Des bibliothécaires sympathiques et compétents de l’Université de Calgary nous ont aidés à construire la plateforme d’une publication en ligne. Ils nous ont guidés tout au long de nos démarches pour faire enregistrer une URL et nous ont offert des fonds de départ en puisant dans une subvention aux publications. Le D^r^Donnon, à titre de corédacteur, moi-même à titre de rédacteur et l’un de nos auxiliaires à l’enseignement à titre de directeur de rédaction, avons lancé la revue. On a annoncé la publication d’une nouvelle revue, lancé un appel à communications, reçu et envoyé des manuscrits pour examen, pris des décisions éditoriales et publié le premier numéro de la revue. La production des numéros suivants a été très exigeante puisque nous n’étions que trois à faire tout le travail. Malgré les formidables défis que représente le démarrage d’une nouvelle revue, on a construit la base, obtenu plus d’aide, persisté et nous y sommes arrivés.

## Partie 2 De 2014 à 2019 (Marcel D’Eon, rédacteur en chef)

Je me souviens de la longue conversation téléphonique que j’ai eue avec le D^r^ Jocelyn Lockyer, en février 2014. Le rédacteur fondateur, le D^r^ Claudio Violato, venait tout juste de quitter l’Université de Calgary pour aller travailler aux É.-U. Le RCÉM était à la recherche d’un rédacteur et personne ne semblait disponible pour prendre sa relève. J’avais été corédacteur du RCÉM pendant un an environ et du *Canadian Journal for the Scholarship of Teaching and Learning* pendant peut-être deux ans. Je n’avais aucune idée de la façon de diriger une revue (qui le sait avant de commencer?). Mais en discutant, je me suis aperçu qu’il serait plus facile de maintenir le RCÉM en vie (même le moindrement) que d’essayer de le faire revivre une fois moribond. Je me suis alors porté volontaire. J’étais courageux, mais naïf. Avec l’aide généreuse de M^me^ Tina Voth, la directrice, du D^r^ Jocelyn Lockyer, vice-doyen principal l’École de médecine Cumming de l’Université de Calgary et la plupart des corédacteurs recrutés, on a poursuivi l’aventure du RCÉM.

Durant les premiers mois, les corédacteurs ont relevé le défi et j’en ai recruté d’autres pour qu’ils nous aident. En décembre 2014, on a réussi de justesse à produire un numéro pour l’année 2014, (nous en avions publié deux annuellement depuis 2010). Nous avons publié deux numéros en 2015, trois en 2016, puis quatre en 2017, 2018 et 2019. Nous avons publié environ 90 pages par année et nous en sommes maintenant rendus à plus de 400 pages par année. Au printemps de 2017, nous avons commencé à placer des images dans chaque numéro, ce qui décore bien nos pages Web. La plupart de ces images représentent des artistes canadiens s’étant illustrés à l’exposition White Coat, Warm (He)Art à la CCÉM.

Lorsque M^me^ Tina Voth a pris sa retraite, nous avons embauché des talents de chez nous. Nous avons été très chanceux de recruter la D^re^ Jennifer O’Brien, qui a accepté de travailler à temps partiel pour nous. Celle-ci est devenue notre directrice de rédaction en 2014. Depuis, elle a été un atout crucial pour le RCÉM. On a embauché une secrétaire de rédaction et une autre personne pour nous aider à suivre la réception d’articles et la revue par les pairs. On a réussi à réduire notre temps de traitement, c’est-à-dire le temps écoulé depuis la première décision suivant la revue par les pairs; il est passé d’une moyenne de plus de 200 jours à bien moins de 100 jours. On a aussi réussi à réduire de moitié le nombre moyen de jours de travail pour produire la publication, qui est passé d’un peu moins de 300 à environ 150, ce qui est un réel exploit, compte tenu du nombre annuel moyen d’articles reçus, qui est passé de 25 à plus de 150, soit six fois plus.

Le RCÉM est redevable envers ses fondateurs. Sans leur soutien matériel, on n’aurait pas pu accomplir ce qu’on a fait et on aurait peut-être été obligés de fermer nos presses. En 2014, l’Université de Calgary nous a versé environ 10 000 $ quand nous nous sommes installés à l’Université de la Saskatchewan. Celle-ci nous a accordé des subventions totalisant 14 000 $ au cours des quatre années qui ont suivi. L’ACÉM a versé au RCÉM plus de 12 000 $ en deux ans. L’AFMC nous a aussi généreusement accordé 5 000 $ pour renflouer nos fonds d’exploitation. Enfin, en mars 2018, le Conseil de recherche en sciences humaines a versé au RCÉM environ 87 000 $ sur trois ans pour couvrir ses frais d’exploitation et permettre la réalisation de projets spéciaux (traduction de résumés et de titres et activités de célébration de notre 10^e^ anniversaire lors de la CCÉM de 2020). D’autres remerciements vont à l’Université de la Saskatchewan, qui m’a permis de toucher un salaire comme de rédacteur en chef du RCÉM. Je suis aussi reconnaissant envers les personnes et les organismes qui ont fait confiance en la revue et qui continuent d’investir du temps et de l’argent dans son avenir.

Nous avons restructuré notre comité de rédaction et recruté de nombreux nouveaux rédacteurs adjoints dans toutes les régions du pays. Ceux-ci représentent 12 facultés de médecine canadiennes réparties dans 13 universités. Ces rédacteurs adjoints aident à faire l’examen préliminaire des articles, à désigner des évaluateurs, à présenter des recommandations à l’éditeur et à communiquer avec les auteurs. Les rédacteurs adjoints constituent le moteur du RCÉM. On était d’avis qu’ils devaient jouer un rôle dans l’élaboration d’une politique éditoriale. C’est pourquoi on les a nommés membres du comité consultatif de rédaction, qui se compose principalement de rédacteurs adjoints.

Au début, les membres du comité éditorial se réunissaient plusieurs fois par année, par téléconférence. Ces réunions étaient importantes et utiles. Notre première réunion en face à face a eu lieu lors d’un midi à Winnipeg le mardi, dernier jour de la CCÉM de 2016. Depuis, on se réunit chaque année à l’occasion de la CCÉM, strictement selon le principe « Apportez votre propre carte de crédit. » Les réunions en face à face sont d’excellents moyens d’assurer une cohésion d’équipe et d’étudier des questions éditoriales importantes.

Parallèlement à la revue et à la publication d’articles (qui sont des activités universitaires malheureusement en déclin pour certaines), un consortium national d’organismes de formation médicale s’est formé et est devenu le conseil d’administration du RCÉM. Des réunions suivies d’autres réunions ont mené à la tenue de consultations. Lorsqu’on est parvenu à un terrain d’entente, on a créé une équipe de cinq partenaires soutenus par des conseillers répartis d’un bout à l’autre du pays et appelés à un rôle de premier plan dans la supervision des aspects commerciaux et financiers du RCÉM. Cette équipe compte des représentants de l’Association des facultés de médecine du Canada, de l’Association canadienne pour l'éducation médicale, du Collège des médecins de famille du Canada, du Conseil médical du Canada et du Collège royal des médecins et chirurgiens du Canada. Les coprésidents sont affiliés à l’AFMC et à l’ACÉM. Nous sommes ravis que les cinq partenaires nationaux qui soutiennent et dirigent le CCÉM chaque année participent activement à la réalisation du RCÉM et le soutiennent.

## Conclusion (D^rs^Violato et D’Eon)

Il fallait créer une nouvelle tribune pour publier et diffuser les travaux de chercheurs et d’universitaires au Canada et à l’étranger. On l’a fait et, comme on l’avait espéré, « ils » sont venus. Le RCÉM a beaucoup évolué depuis ses débuts. De nombreuses personnes ont dû travailler d’arrache-pied pour nous amener jusqu’ici, plus de 10 ans plus tard. Mais il faudra encore plus de bras pour nous permettre d’avancer, à mesure que nous deviendrons un partenaire essentiel de la formation médicale au Canada, et par la suite, évidemment, dans le monde entier.
